# A Machine Learning Assisted, Label-free, Non-invasive Approach for Somatic Reprogramming in Induced Pluripotent Stem Cell Colony Formation Detection and Prediction

**DOI:** 10.1038/s41598-017-13680-x

**Published:** 2017-10-18

**Authors:** Ke Fan, Sheng Zhang, Ying Zhang, Jun Lu, Mike Holcombe, Xiao Zhang

**Affiliations:** 1 CAS Key Laboratory of Regenerative Biology, Joint School of Life Sciences, Guangzhou Institutes of Biomedicine and Health, Chinese Academy of Sciences, Guangzhou 510530, China;Guangzhou Medical University, Guangzhou, 511436 China; 2 Guangdong Provincial Key Laboratory of Biocomputing, Guangzhou Institutes of Biomedicine and Health, Chinese Academy of Sciences, Guangzhou, 510530 China; 3 Department of Computer Science, University of Sheffield, Sheffield, United Kingdom; 4 epiGenesys, Sheffield, United Kingdom

## Abstract

During cellular reprogramming, the mesenchymal-to-epithelial transition is accompanied by changes in morphology, which occur prior to iPSC colony formation. The current approach for detecting morphological changes associated with reprogramming purely relies on human experiences, which involve intensive amounts of upfront training, human error with limited quality control and batch-to-batch variations. Here, we report a time-lapse-based bright-field imaging analysis system that allows us to implement a label-free, non-invasive approach to measure morphological dynamics. To automatically analyse and determine iPSC colony formation, a machine learning-based classification, segmentation, and statistical modelling system was developed to guide colony selection. The system can detect and monitor the earliest cellular texture changes after the induction of reprogramming in human somatic cells on day 7 from the 20–24 day process. Moreover, after determining the reprogramming process and iPSC colony formation quantitatively, a mathematical model was developed to statistically predict the best iPSC selection phase independent of any other resources. All the computational detection and prediction experiments were evaluated using a validation dataset, and biological verification was performed. These algorithm-detected colonies show no significant differences (Pearson Coefficient) in terms of their biological features compared to the manually processed colonies using standard molecular approaches.

## Introduction

Stem cells are a kind of self-replenishing cell population. Their primary function is to generate progeny, which then develop into terminally differentiated cell types. Tissue-specific adult stem cells or progenitors are committed to producing tissue- or lineage-specific cells. Other kinds of stem cells called pluripotent or even totipotent stem cells have the potential to give rise to any of the 200+ cell types in all three germ layers. The following are the two types of pluripotent stem cells defined by their tissue origin: (i) embryonic stem (ES) cells obtained from early embryos, typically at the blastula stage and (ii) induced pluripotent stem (iPS) cells derived through a reprogramming process whereby terminally differentiated somatic cells are reprogrammed or induced back to a pluripotent state. iPS technology is a personalized approach and does not require embryos; therefore, unlike ES cells, the ethical issues do not apply. Recently, iPSCs have been used to treat chronic and degenerative diseases; for example, retinal degenerative blindness due to retinitis pigmentosal^1^. Thus, clinical applications of iPS cells can be generated for personalized formats for cell replacement therapy. Moreover, these are strong demands for quality-assured iPSC lines from both pharmaceutical industry and basic medical research organizations^2^. A non-invasive approach has been established to convert urinal cells into iPSCs. This is the most convenient way to obtain a cell source for reprogramming studies, and we have implemented this method in this study for data training and statistical modelling^3^.

One issue that appears to limit iPSC quality or biological consistency in further applications is colony determination, which leads to the isolation and purification of iPSC colonies during the cellular reprogramming process. The current solution relies on a judgement call from well-trained cell culture experts, though the training is time consuming and has a high cost. In contrast and inevitably, the natural instability of human recognition will could allow for misjudgement, which can cause batch-to-batch variation. The quality control of iPSC colony determination is extremely important for downstream expansion and for maintaining a homogeneous culture of undifferentiated cells. Inconsistency in colony determination and selection will cause insufficient downstream differentiation into functional cells. Therefore, an automated quantitative method with consistent colony maturation determination will be a great for assisting biologists during the iPSC formation process.

The traditional approach for identifying and verifying iPSC colonies is to use immunofluorescence staining or a reporter system to detect pluripotent markers, which could couple well with automated fluorescence microscopy provides and provide a dynamic and effective method^4,5^. However, this kind of xeno probe-based labelling can only be applied during a late stage in reprogramming; this cannot be delayed further due to potential safety issues for downstream clinical applications^6,7^. Furthermore, during cellular reprogramming studies, it has been observed that along with positive iPSC colonies, negative colonies contain various morphology subtypes, especially with respect to cellular polarity. The cellular polarity is strongly linked to the specificity of gene expression, cell cycle, and other cellular regulation and may explain the different reprogramming mechanisms^8^. For example, the cell polarity changes between the mesenchymal-to-epithelial transition (MET) or EMT, which is linked with induced pluripotent stem cell (iPSC) colony formation or tumour genesis^9–11^. Hence, the reprogramming process has a strong link with morphology changes; the classification of the morphology can be used as a read-out in a quantitative way to identify iPSC colonies and to monitor the reprogramming process. This provides richer information than the binary fluorescence images and opens the door for a label-free, non-invasive approach.

Here, we report a label-free, non-invasive and consistently quantitative method for human iPSC detection in an accurate way. This automated system involves a computer vision recognition model to assist in the classification of iPSC colonies from bright-field microscopic images. This model utilizes a convolution neural network as a classifier in a sliding windows framework for colony recognition. Subsequently, a semi-supervised segmentation method was applied to locate the colonies and detect their boundaries. Moreover, a Hidden Markov Model (HMM) was trained to estimate the growth phase and maturation time window of colony formation during the reprogramming process. Finally, using data from colonies traced via time-lapse, this system can predict the best selection time window for iPSC colonies to prevent random differentiation caused by overgrowth. Our results show that this algorithm detected and predicted colonies and demonstrated no significant differences (Pearson coefficient r > 0.9) in terms of biological features compared to manually processed colonies. This was evaluated and characterized biologically using standard immunofluorescence staining, quantitative polymerase chain reaction (QPCR), and RNA-Seq for pluripotent verification.

## Results

### Automated iPSC colony detection using computer vision algorithms and verified via OG mice as Oct-4-GFP reporter system

**Figure 1:**
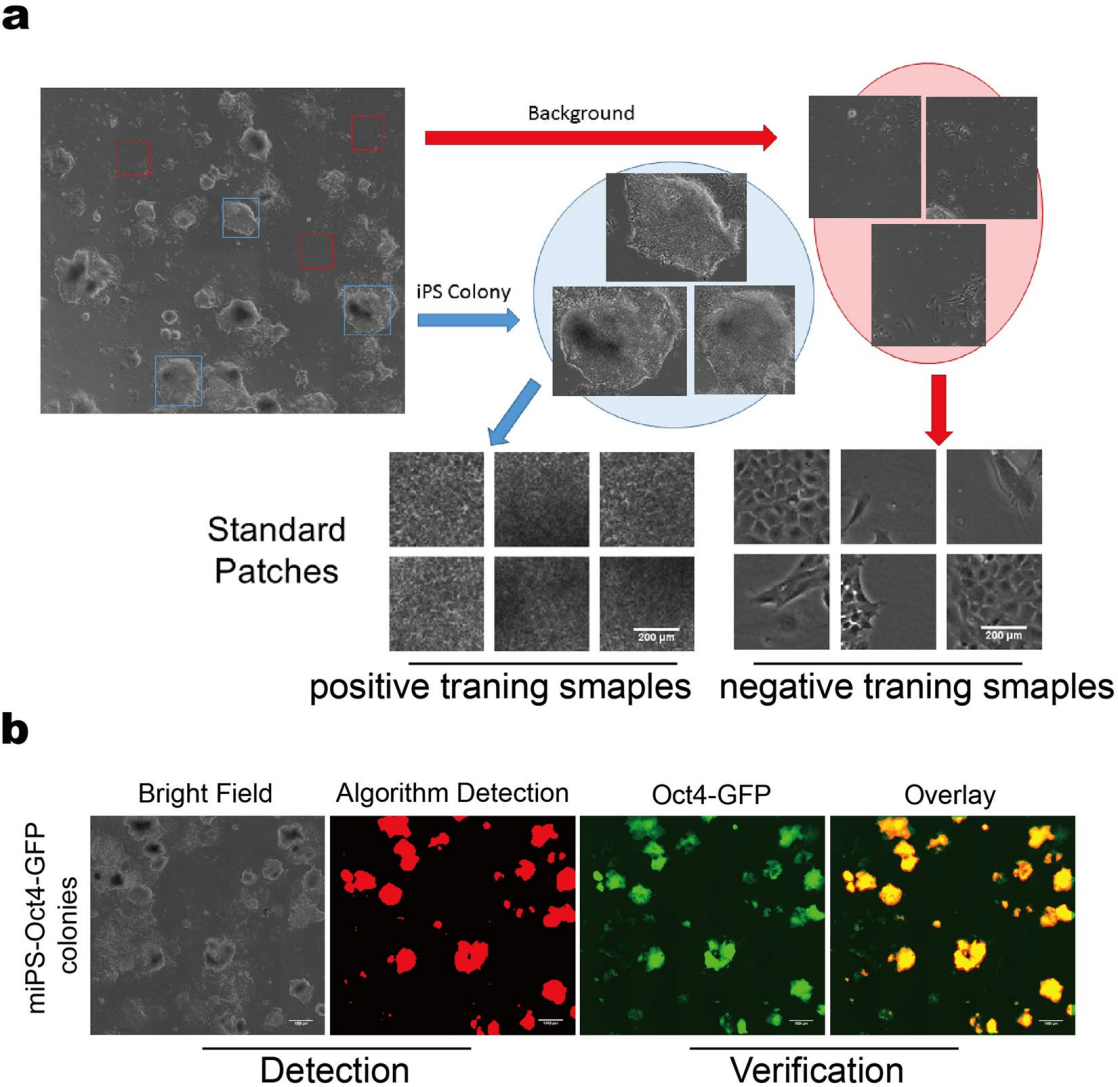
Computer vision based detection of mice iPSC colonies. (**a**) Example of training patches. Positive samples cropped out as iPSC colonies and negative samples are MEF cells or background. (**b**) Verification of computer vision based method using the OCT4-GFP reporter. Panels from left to right indicate the bright field original image, a mask of computer detection, an OCT4-GFP fluorescent image, and an overlay between the fluorescence and binary images.

During the reprogramming process, the cellular polarization reshaping leads to morphology changes, which indicate iPSC colony formation. A novel computer vision assisted method was developed to process the microscopic images. The sliding window approach was performed to scan the potential colony areas, followed by the detection, which generates a binary image. Post-processing including discarding the fragmented areas was performed using the binary images as the template. Therefore, the areas of interest referring to the iPSC colonies can be determined based on size and location (Fig. 1b). Examples of cropped positive and negative colony texture mosaic windows from the algorithm training is shown in Fig. 1a. The algorithm detects the positive areas in a whole well from a 6-well cell culture plate.

The merits of the algorithms were assessed by verifying the biological signature of the iPSCs using OG mice with an OCT4- GFP knock-in reporter mice embryonic fibroblast (MEF) system. The activation of the OCT4 pluripotent gene indicated MEF reprogramming success of iPSCs^12^. This biological fluorescence indication was used as a positive feedback mechanism for algorithm training. Subsequently, we used the trained classifier to generate the isolated binary images, where the fluorescence signal areas were masked out as the overlay [Fig. 1b]. The observations showed that nearly 10% of the cells from the total population did not express OCT4-GFP; thus, these cells were not selected by the algorithm. The quantitative analysis showed that the correlation between OCT4-GFP expression and the algorithm overlay was highly relevant, with an average Pearson’s Coefficient of r = 0.877. The r-value was determined from 3 individual reprogramming experiment data sets (other results are shown in Supplemental Figures S2 and S3).

### Human iPSC colony detection using computer vision based algorithms verified via pluripotency biomarkers

**Figure 2:**
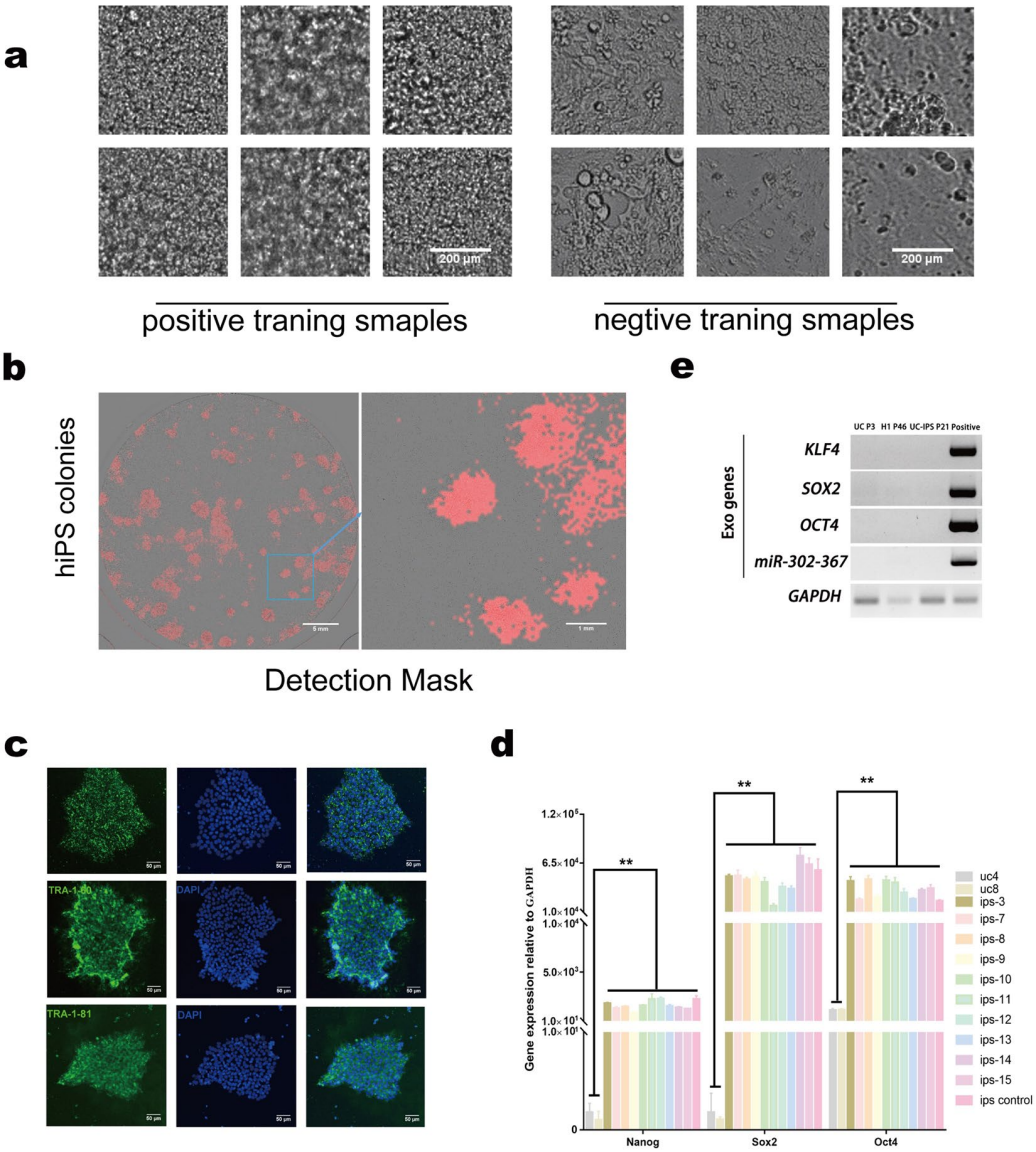
Computer vision based detection of human urinal cells (UCs) derived iPSC colonies. (**a**) Example of training patches. Positive samples are parts of iPSC colonies, and negative sample are other cells or background. Scale bar, $200\mu {\rm{m}}$. (**b**) A detection map. Example of iPSC colonies detection in one 6-well plate at Day 21 after reprogramming induction. Red regions indicate the position of iPSC colonies, which were detected by bright field image classification algorithm. A small area is enlarged to show the details of the detection. (**c**) Characterization of iPSCs, which were manually picked guided by the binary map. iPSC colonies (n = 3) were picked into cell culture plate based on the binary map. After 4–7 days culturing, iPSCs were stained with antibodies against known pluripotency surface markers (left). The nucleus was stained by DAPI (middle), merged images of two channel (right). Scale bar, 50*μ*m. (**d**) Selected iPSCs were analysed for Nanog, SOX2 and OCT4 expression by qRT-PCR. Human ES cell line H1 was used as positive control. Error bars indicate Â ± s.d. of triplicates. *P* value is referenced to UCs. **Indicates *P* < 0.01. (**e**) PCR detection of exogenous eipsomal DNA in UC-iPSC. UC(Passage 3) and H1(Passage 46) cell were severed as the negative control, UC-iPSC(Passage21) was stably expanded, UC transiently transfected with eipsomal vector (pEP4EO2SET2K and pCEP4- miR-302-367 cluster) was served as the positive control.

Human iPSC regeneration opens the door as a personalized cell source for cell replacement therapy in regenerative medicine. This algorithm has extended the non-invasive and label-free computer assisted method for human urinal cell reprogramming^3^. Different from the OG mice system, since urinal cells OCT4-GFP is not applicable in this case, we built the training dataset using manual cropping processed by well-trained cell biologists (Fig. 2a). As shown in the previous section, our iPSC detection framework consists of a mosaic sliding window-based classification process. If a small patch is located in a positive iPSC colony texture, the area was then masked out by the overlay; otherwise, it is a negative patch. The training session was run with numerous manually cropped samples, followed by the annotation of the positive colonies; this indicated the locations of regions after post-processing for an entire well in a 6-well plate (Fig. 2b).

This computer-guided selection was implemented after combining the generated binary image with the bright-field image, and the colony-picking decisions were made only based on the computer vision results. Subsequently, to verify the computer-guided iPSC colony detection, manual picking was performed, followed by standard iPS cell characterization (Fig. 2c,d). The expanded colonies that passed the characterization, including karyotyping by G-band analysis (data not shown), were further characterized for pluripotency. Here, we show iPSCs generated from sample c4p2. For example, fluorescent immunostaining was initially performed to demonstrate the expression of pluripotent surface markers, such as TRA-1-60, TRA-1-81 and SSEA4 (Fig. 2c). Then, the endogenous pluripotent genes, such as OCT4, SOX2 and NANOG, were fully activated, and were comparable to human embryonic stem line H1 cells (Fig. 2d). Finally, using genomic PCR, it was confirmed that the UC-iPSCs expanded at passage 21 and no longer harboured the exogenous reprogramming factors from the original episomal plasmid (Fig. 2e). Hence, this computer vision approach worked for both human iPSC detection as well as in the mouse model.

### Quantitative detection of synchronized time-lapse data for iPSC selection prediction in human somatic cellular reprogramming

The somatic cellular reprogramming was indicated by cell polarization changes in morphology. Based on experience, urinal cell reprogramming systems take more than 17 days to reach the matured and complete stage. The computer vision machine learning method allowed us to determine the stage of cellular reprogramming, and this was not limited by the experienced developed criteria. To monitor the complete reprogramming in a quantitative manner, new sets of time-lapse training data were used to model iPSC formation and to classify the phases in this process.

**Figure 3:**
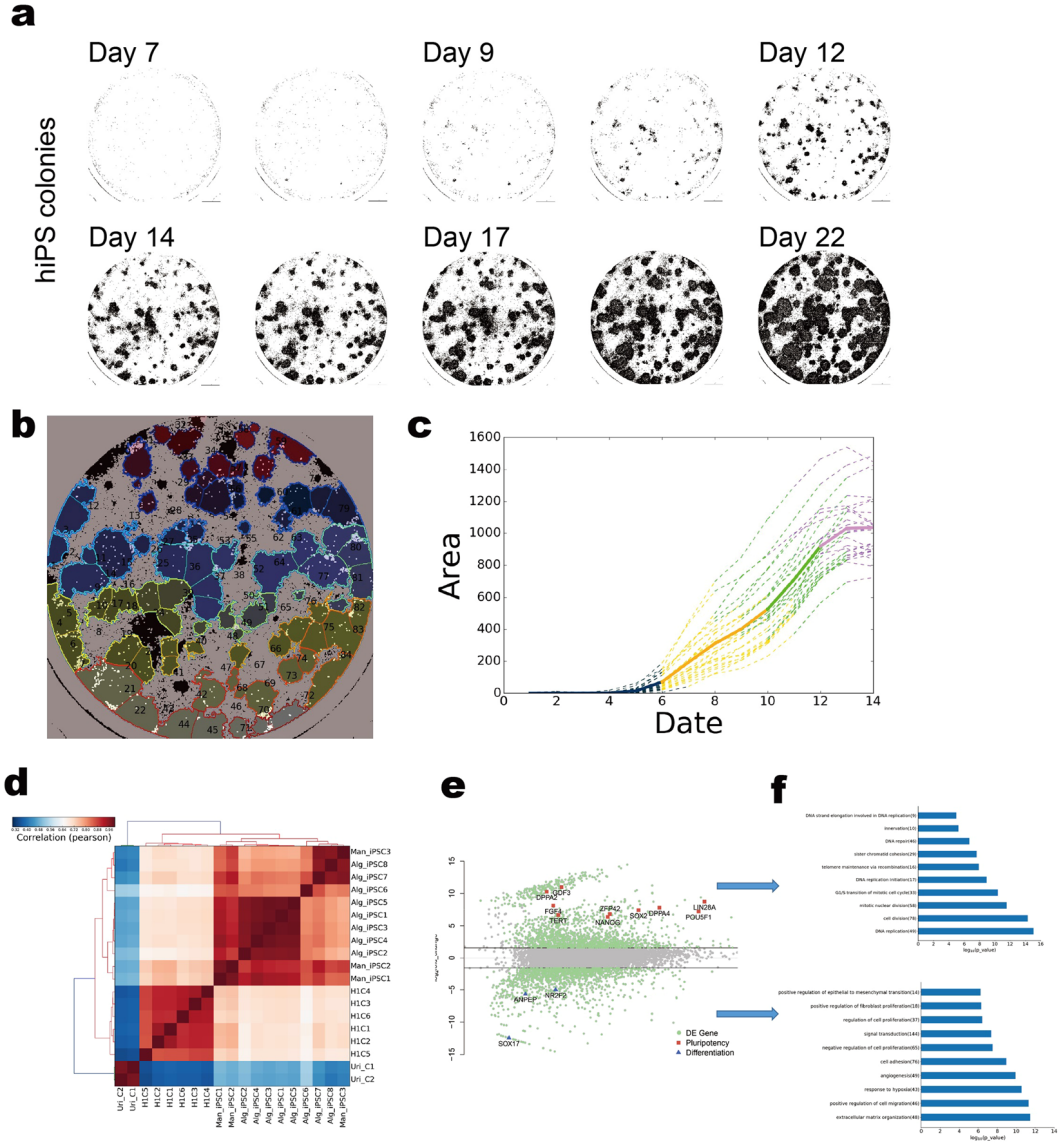
Quantitative detection in synchronized time-lapse data for iPSCs selection prediction in human somatic cellular reprogramming. (**a**) A series detection map for the entire reprogramming process from day 7 to day 22. Binary indication masked out the colonies by classifier. (**b**) A segmented map. An example result of segmentation on a detection map, each segment represents a single colony (left); each colonies was individually registered for further process(right). (**c**) Growth curve of selected colony. Different colour indicted for different phases, which was calculated by the mathematical model; the purple colour stands for the mature phase. Solid line is an average of all training data and dash lines are some examples of training data. (**d**) Hierarchic clustering analysis of global gene expression based on Pearson correlation for Alg_iPSCs, H1 (C1–6) and Uri_C(1–2). (**e**) Differential gene expression profile between Alg_iPSCs (above) and UCs (below). The differentially expressed genes (red) are those with an adjusted P value 0.05 and fold change 3. (**f**) Functional annotations of genes differentially expressed between Alg_iPSCs (top) and UCs (bottom). Gene ontology (GO) was performed by DAVID, and enriched GO terms (biological processes) for each cell type are plotted with −*log*_10_ of the adjusted P values.

First, an individual colony was back traced as described early on. Combined with the detection method, the earliest feature for cellular polarity changes, the formation of the iPSC texture can be recognized at day 7 after reprogramming induction. An example of the detected time-lapse data is shown in Fig. 3a. Subsequently, the individual iPSC texture features were detected using a segmented boundary, and each feature was registered and tagged digitally (Fig. 3b). Each iPSC texture feature was monitored individually during the entire reprogramming process. We can easily exclude the over-grown and under-grown colonies using the average growth rate. This gives a quantitative measurement for colony formation, which is shown in the growth phases, and each curve line represents a qualified iPSC clone (Fig. 3c). Moreover, a Hidden Markov Model (HMM) with four quantitative features was applied to analyse this process. This means that under the comparable reprogramming conditions with the same cells types, the model classified four different phases in the growth curve to describe the characterization of the iPSC clone forming mathematically. Finally, this model gives a posterior probability for each phase; for example, between days 12 and 17, the probability score of maturation phase increased from 0 to 1. The model provides the correlation between the iPSC harvest time and the probability score. The image data for best picking decision was selected manually and fed into the model to calculate the selection threshold. The probability score of 0.3 was the output to describe the picking threshold in the urinal cell reprogramming system. For example, in this data set, the algorithm gives the closest score to 0.3 on day 14, which means that colony picking could be triggered. Therefore, the optimal picking time window can be predicted. If the probability factor reaches 1, this means the cells are overgrown, which implies a risk of random differentiation.

In separate experiments, iPSC colony detection was synchronized using a clone-by-clone approach, and the colony selection was triggered as described above. Subsequently, these colonies were verified using a sample enriched for high-throughput RNA-Seq gene expression analysis. The pluripotency- and germ layer-specific marker genes were plotted for comparison.

We analysed the global gene expression patterns from the algorithm detected in urinal cell-derived iPSCs (Alg_iPSCs), UCs and the embryonic stem cell H1. A hierarchical clustering heat map (Fig. 3d) showed that the gene expression in the Alg_iPSCs is highly independent from the UCs. Comparison of the expression profiles between the UCs and Alg_iPSCs showed that 1759 genes were up-regulated in the Alg_iPSCs, while 1538 genes were up-regulated in the UCs (Fig. 3e).

The Gene Ontology project provides a controlled vocabulary to describe the differentially expressed genes. The up-regulated genes in the UCs were enriched in GO terms related to epithelial, angiogenic or fibroblast function. In contrast, the up-regulated genes in the Alg_iPSCs were related to DNA replication and mitotic division. These GO terms also strongly demonstrated the differentially expressed genes between the H1 cells and UCs (Supplement data Figure S4). Further analysis revealed that the pluripotency genes were more highly expressed in both UC_iPSCs and H1 cells compare to their low expression in the UCs. The data showed no distinctive expression between the Alg_iPSCs and Man_iPSCs or between the Alg_iPSCs and the H1 cells. In contrast, the UC gene expression profile is clustered in a totally different manner.

## Discussion

Recent developments in computer vision techniques can provide advance tools for cellular morphology description and classification^4,13^. The application of a computer vision system provides quantitative analysis in cell biology, which can reduce human error and improve consistency^14–20^. Historically, digital pathology has primarily focused on low-level image feature extraction^21–23^ (e.g., colour and intensity, nuclear segmentation, and typical morphology features), followed by the construction of classification models using classical machine learning methods, including: Support Vector Machines^24,25^ and Random Forests^26^. Manual design and the selection of features is based on human observations and experiences, which make it hard to achieve optimal performance. Moreover, normal classifiers are designed to suit relatively small sets of training data.

Deep learning has recently attracted attention in the machine learning field. The motivation is to use big data to directly train multi-layer neural networks with different deep structures and combine these networks with feature extraction and classification. Convolution Neural Network (CNN) is a structure introduced by LeCun^27^ that is widely used in computer vision area. Its most successful application is dealing with image data in classification, segmentation detection and retrieval task. For example, CNN is used as the current baseline approach in breast cancer classification and diagnosis^28^. Chen *et al*.^29^ present a multi-feature, label-free cell classification system using deep learning techniques. The beauty of the deep learning technique is the end-to-end learning procedure that combines feature extraction with the classifier. The performance will be benefited by expanding training data set.

Here, we present a machine learning-based system for the automated detection and prediction of iPSC formation using a cellular reprogramming process. This model works in both mice and human reprogramming systems, and the HMM-based model was used for phase probability estimation to trigger iPSC colony selection. Our approach, which uses a Deep Convolution Neural Network (DCNN) end-to-end learning framework, can avoid the non-optimal manual design of extractors and classifiers when faced with complicated cell textures and morphology changes, which provides optimized performance and convenience.

In the application point of view, this computer-aided machine learning approach for iPSCs can detect the cells earlies and predict the optimal selection time. This means that this tool can quantitatively evaluate different somatic reprogramming approaches, such as using engineered transcriptional factor or small molecules, for iPSC generation or further differentiation. If the time-lapse data has a high frequency frame rate, this automated high-throughput analysis could potentially group the different stages as individual conditions for cellular reprogramming for cell fate mapping modelling.

Finally, the whole reprogramming process is serum-free, feeder-free and uses episomal based induction, this computer vision guided label free and non-invasive approach was fully verified by standard biological approaches, as well as RNA sequencing. We expect these combined approach will become an everyday technique for cell biology studies in a quantitative way. It should not limited in cellular reprogramming works. This system can be developed further includes to study downstream cell differentiation, and cell line development to identify appropriate cells in a fully traceable and quantitative way, or even guide an automated robotic in application of regenerative medicine.

## Methods

### Ethic Statement

*Mus musculus* (mice model) were maintained and cared for in our Experimental Animal Centre’s facility in accordance with the Guangzhou Institutes of Biomedicine and Health Institutional Animal Care and Use Committee (approval number N2017039) protocols. All experiments were performed in accordance with the guidelines set by the Human Subject Research Ethics Committee at Guangzhou Institutes of Biomedicine and Health (GIBH) and the Chinese Academy of Sciences (CAS), and the Committee approved the experiments (approval number GIBH-IRB07-2017039). Formal informed consent was obtained from all the subjects.

### Overall Computer-Assisted System

**Figure 4:**
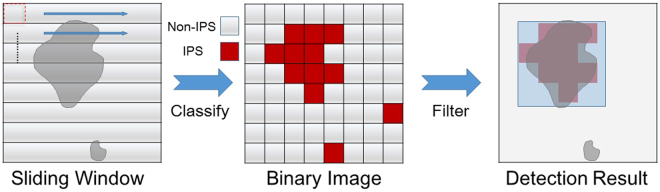
The sliding window method was used to detect colonies from an unknown image. The small areas were divided into 96 × 96 pixel patches.

The iPSC colonies in the bright-field microscopy images normally represent different morphology features from other cells. However, it is difficult to detect single cells in this image, because the cell edges and boundary overlap and are fuzzy. We did not adopt a traditional segmentation and recognition procedure. Our strategy was to identify a typical colony texture rather than a single cell feature. We used the sliding window method to detect the potential colony areas (Fig. 4).

A 96 × 96 patch was defined as a fixed detection window, specifically a standard patch from the top left corner “slides” across the whole testing image. For each of these windows, we took the window region and applied CNN to determine whether the patch is an iPSC area or not. Finally, a binary map is obtained that indicates the locations and sizes of the iPSC colonies in the testing images. Every input image used in our system has to be normalized to reduce the influence from illustration conditions. Because the background is dominated by the brightness of the entire image, we can assume that once the brightness is normalized, the differences between images will be minimized. In our recognition system, the pre-processing process is a modified Autolevels (AL) algorithm (Supplemental data 1.1) that normalizes brightness and enhances the acquired images. In this study, CNN showed the best recognition rate compared to other methods (Supplemental data 1.2).

After the binary image was created, the morphology transformation was applied; this transformation filled the holes. We ran a Gaussian filter and performed a re-binary method on the image using a certain threshold to remove the single noise points. Utilization of the GFP reporter as the positive labels in the mice data allowed us trained a good classifier, which allowed us to directly identify contours and boundaries from the binary image. Finally, the image was processed to filter out any sparse residuals that counted as noise.

To locate human urinal cell-derived iPSC colonies and their boundaries, the initial data collection was selected for day 12, which is a seed frame for segmentation. For the data before this decided time point, a positive binary window of points was counted as the area in the contours of certain colonies. In more matured cases, a semi-supervised Random Walker algorithm^30^ was implemented to expand the area to complete the boundaries and used labels to mark the detection. An example of segmentation on day 20 is presented in Fig. 3b.

### Growth Curve Modelling

After the boundary of a colony was found, each colony can be measured in terms of the area and time frame after reprogramming induction. A growth curve can be plotted based on the area of detected signals versus time after reprogramming induction (Fig. 3c). Abnormal growth conditions (overgrowth/undergrowth) were manually defined by a stem cell specialist after reading through the all the acquired image files after the reprogramming process. Subsequently, the abnormal phase was discriminated based on the mean of first-order differences in the growth curve between days 10 and 20. The remaining 97 normal samples were used to train the HMM model for prediction using the following four features: (1) the first order difference in the growth rate, (2) the second order difference in the growth rate, (3) the area, and (4) the number of growth days. Two stages are labelled to find the best fit HMM model. The human experts labelled the days for the best picking time for every colony. The period from one day ahead of the picking day to the end is labelled the mature stage. The period from day 1 to two days after the positive sample is shown is labelled first stage. We can use the Baum–Welch algorithm to train the HMM model with 2000 different initializations. The best model can fit the labelled data with 4 hidden stages, and the middle part is divided into two periods of similar length. Finally, the Viterbi algorithm^31^ can be used to predict the hidden stages of growth curves, of which an example is shown in Fig. 3c.

### Image and Dataset Acquisition

The MEF cells were cultured and imaged via Zeiss Z1 microscopy. The image is of a certain area and each well was captured every 2 hours. The human cells in each well were imaged with the Solentim Cell Metric once each day. These images were acquired at a 16-bit intensity depth with a MEF resolution of 3322 × 2496 for each region and the human cells were acquired at 17702 × 17684 for each well. More than 50 images for both cell types were acquired from 3 different plates for training. Biologists manually annotated the iPSC colonies and cropped hundreds of iPSC areas and a considerable number of negative samples from the acquired images. Notice that the cropped images had various widths and heights ranging from 200 to 400. In each selected area, a 96 × 96 pixel bounding box was randomly placed and cropped to a standard patch. A class label 0 is assigned to the feature vector of each iPSC, while a class label 1 is assigned to each non-iPSC. We finally obtained more than 2 × 10^6^ standard patches for the two cell types, which included both iPSCs and non-iPSCs. A repeated random sub-sampling evaluation (Supplement data 1.2) on this dataset showed that the CNN achieved the best accuracy for our study.

### Neural Network Design and Iterative Training

We used a modified AlexNet^32^ structure as the base of our neural network design. Because our colony detection application is a binary classification problem, the number of network sizes can be shrunk to a smaller scale without a performance drop. A batch-normalized layer was added before each convolution layer to accelerate the training speed and slightly improve the performance. To fine-tune the performance of our network, biologists will check the automatic recognition results for a new capture image and mark incorrect regions after the initial training. The incorrect data will add to the training sets and be corrected with the right label. After more than 3 fine-tuning processes, the detector will achieve a nearly human level recognition rate for iPSC colonies.

### Mice embryonic fibroblast cell reprogramming

According to the previous OKS protocol^33^. Oct4-GFP transgenic MEFs were plated at 4000 cell/cm2, and then transfected with retrovirus(retroviral vectors carrying murine cDNAs for Oct4, Sox2, Klf4 and c-Myc were purchased from Addgene.) using packed plat-E cell. Forty-eight hour post-transfection, the viral supernatants were removed, culture medium was added and time point was defined as day 0. iPSC-inducing medium contained DMEM+vitamin C, bFGF, CHIR99021 and other chemical components. Photos of cells in the well were taken by cell metrics or with a SteREO Lumar.V12 (Zeiss) every day.

### Human urinal cell culture and reprogramming

Urine cell (UC) collection, culture and reprogramming were performed as described in Xue *et al*.^3^ with some modification. Briefly, the primary urine cells were cultured in urine cell medium consisting of an equal proportion of DMEM/F12 mixed with MEF medium containing 10% foetal bovine serum (FBS, PAA), 0.1 mM NEAA (Gibco), 1 mM L-glutamax (Gibco) and a SingleQuot Kit CC-4127 REGM (Lonza). The 1–2 × 106 urine cells were segregated with 0.25% trypsin treatment. After centrifugation, 6 µg of the T2k vector and 4 µg of the microRNA302 vector were co-transfected into the UCs using an Amax™ Basic Nucleofector™ Kit (Program, T-020, LONZA) according to the manufacturer’s instructions. Transfected UCs were grown on matrigel-coated P6 wells in UC medium. After 24 hours, the UC medium was replaced by TESR supplement with 4i (4i:CHIR99021 (3 µM), A83–01 (0.5 µM), thiazovivin (0.5 µM), and PD0325901 (1 µM)). After 12 days, the cocktail of four small molecules were removed. Bright-field images were taken by Cell Metrics every day and were analysed by our detection system. The system detected and indicated the mature iPSC colonies around the 25th day. Colonies were manually marked at their corresponding location, picked, and plated on matrigel-coated 96-well plates containing mTesR medium.

### Immunocytochemical and Quantitative RT-PCR (qRT-PCR) analysis

Immunofluorescence was used to characterize the iPS cells as previously described (Anti-TRA-1–60, Millipore; Anti-SSEA4, Life Technologies). DAPI was used to stain the cell nuclei. Fluorescence-labelled cells were imaged on The ImageXpress® Micro Confocal High-Content Imaging System. Total RNA was extracted using the RNeasy Mini kit (Qiagen Cat No. 74104). First-strand cDNA was synthesized from 1 µg of total RNA using the GoScript^TM^ Reverse Transcription System (Promega Cat No. A5000) and qRT-PCR was performed on a CFX96 machine (BIO-RAD) and performed with three biological replicates by the using SYBR PCR Kit (SsoAdvancedTMUniversal SYBR®Green, Cat No. 1725272). The CT method was used to calculate the relative gene expression level. The primers used for genomic PCR and QPCR are listed in supplemental table (Supplemental Table S2).

### RNA Sequencing

RNA sequencing was performed using a protocol in a previous publication^34^. After recognizing and picking the iPS clones, the iPS clone cells were lysed with 200 µl of Trizol (Invitrogen). Total RNA was prepared with the Direct-zol RNA MiniPrep kit (Zymo Research). RNA was then quantified, purified and used to generate cDNA sequencing libraries using the TruSeq RNA Sample Prep Kit (Illumina). The Qubit dsDNA HS Assay kit (Invitrogen) was used to detect the cDNA library concentrations. Sequencing was performed on a MiSeq system with MiSeq Reagent Kits v2 (50 cycles) (Illumina).

## Electronic supplementary material

Supplementary Data
