# Supplementary material for: A Machine Learning Assisted, Label-free, Non-invasive Approach for Somatic Reprogramming in Induced Pluripotent Stem Cell Colony Formation Detection and Prediction
Source: Sci Rep. 2017 Oct 18;7:13496. doi: 10.1038/s41598-017-13680-x (PMC5647349; doi:10.1038/s41598-017-13680-x)
Supplement: Supplementary file 1 — Supplementary Data [file 41598_2017_13680_MOESM1_ESM.pdf]

Machine Learning Assisted Label-free and Non-sinvasive Approach for Somatic Reprogramming in Induced Pluripotent Stem Cell Colony Formation Detection and Prediction

Ke Fan[1,2,+], Sheng Zhang[1,2,+], Ying Zhang[1,2], Jun Lu[1,2], Mike Holcombe[3,4], and Xiao Zhang[1,2,*]

[1]CAS Key Laboratory of Regenerative Biology, Joint School of Life Sciences, Guangzhou Institutes of Biomedicine and Health, Chinese Academy of Sciences, Guangzhou 510530, China;Guangzhou Medical University, Guangzhou 511436, China.
[2]Guangdong Provincial Key Laboratory of Biocomputing, Guangzhou Institutes of Biomedicine and Health, Chinese Academy of Sciences, Guangzhou 510530, China
[3]Department of Computer Science, University of Sheffield, Sheffield, United Kingdom
[4]epiGenesys, Sheffield, United Kingdom
[*]zhang_xiao@gibh.ac.cn
[+]these authors contributed equally to this work

1 Supplementary Information

1.1 Pre-processing

Almost all acquired images under bright-field microscopy had limited dynamic range and variant brightness. In order to improve the iPSCs detection, image enhancement algorithms were applied. Traditional automatic image enhancement algorithms, such as histogram equalization or homomorphic filtering can either enhance or normalize the acquired microscopic images. In this study, we modified the automatic contrast stretch algorithm commonly referred as Autolevels (AL), which normalize the brightness and contrast of an image by remapping the dynamic range of the data to match up with the dynamic range of the display, thus utilization of AL eliminated the outliers in the histogram of the image. The Autolevels algorithm is an image enhancement via the 2-parameter $(low, high)$ gray level transformation defined by

$$\begin{aligned} \text{for } (i &= 0;\ i < L;\ i{+}{+}) \\ T[i] &= \text{Quantize}\left(L\frac{l - low + b}{high - low}\right); \end{aligned} \tag{1}$$

where $0 \leq low < high \leq L-1$, l is the gray level of the original image, b is the brightness bias to the standard (here we choose $L/2$) and L is possible gray levels per pixel, e.g., $L = 256$ for 8-bit image and $L = 65536$ for 16-bit image. And,

$$\text{Quantize}(x) = \begin{cases} 0 & x < 0 \\ \lfloor x \rfloor & 0 \leq x < L \\ L-1 & x \geq L \end{cases} \tag{2}$$

where $\lfloor \cdot \rfloor$ denotes the Floor function, and low and $high$ are two extreme values, which control the amount of gray level clipping. That is, the user provide two small, positive real-valued parameters, C_{low} and C_{high},

Table S1. Recognition result when using different features on mouse dataset.

repeat #		1	2	3	4	5	average
Zernike	SVM	94.4%	87.3%	94.5%	94.7%	89.3%	92 %
	DT	89.3%	95.1%	95.2%	90.1%	95.1%	93 %
	RF	95.4%	95.1%	87.7%	95.2%	95 %	93.7%
Wavelet	SVM	94.4%	89.8%	94.6%	94.7%	90.3%	92.8%
	DT	88.7%	95.0%	95.2%	90.4%	95.2%	92.9%
	RF	95.3%	95.1%	88.6%	95.1%	95.0%	93.8%
Gabor	SVM	97.1%	97.5%	97.7%	97.1%	97.4%	97.4%
	DT	98.9%	97.4%	98.1%	98.8%	97.0%	98.0%
	RF	97.8%	98.6%	97.2%	97.9%	98.7%	98.0%
Combined	SVM	97.6%	97.3%	98.8%	96.8%	98.1%	97.7%
	DT	98.2%	98.3%	97.6%	98.1%	99.6%	98.3%
	RF	99.3%	97.6%	98.5%	99.4%	97.0%	98.4%
CNN		99.9%	99.8%	99.9%	99.9%	99.1%	99.7%

$(C_{low} + C_{high} < 1)$ that specify the amount of low-end and high-end clipping desired respectively. Typical values for C_{low} and C_{high} are 0.005 in which case approximately 0.5% of the image pixels will have their value clipped to black and another 0.5% will be clipped to white. The two algorithm parameters, *low* and *high*, are then determined by solve the two equations

$$\begin{aligned} P[low] &= C_{low}, \text{ and} \\ 1 - P[high-1] &= C_{high}, \end{aligned} \tag{3}$$

for *low* and *high* respectively where $P[\cdot]$ is the cumulative gray level distribution of a being processed image.

1.2 Feature and classifier evaluation

The experiments were performed in a repeated random sub-sampling validation protocol on mice dataset, with the GFP signal (as positive label) obtained from the activation of OCT4 pluripotent. All patches were randomly split into half training and half validation data, and repeated this process randomly for 5 independent cycles. We evaluated the performance of different features and classifiers. Three types of feature (Zernike, Wavelet and Gabor) were trained with Support Vector Machine (SVM), Decision Tree (DT) and Random Forest (RF) classifier respectively. The linear kernel was chosen for training SVM, and values of C was set to be 100 which provide better results. The maximal depth for Decision Tree was set to be 5 in training DT. RF is trained to construct 30 trees containing maximal 9 features. Combined features include all three types were tested as well. All results of recognition accuracy is shown in Table S1. Combination of three features can improve the recognition performance, which is still lower than Convolution Neural Network method.

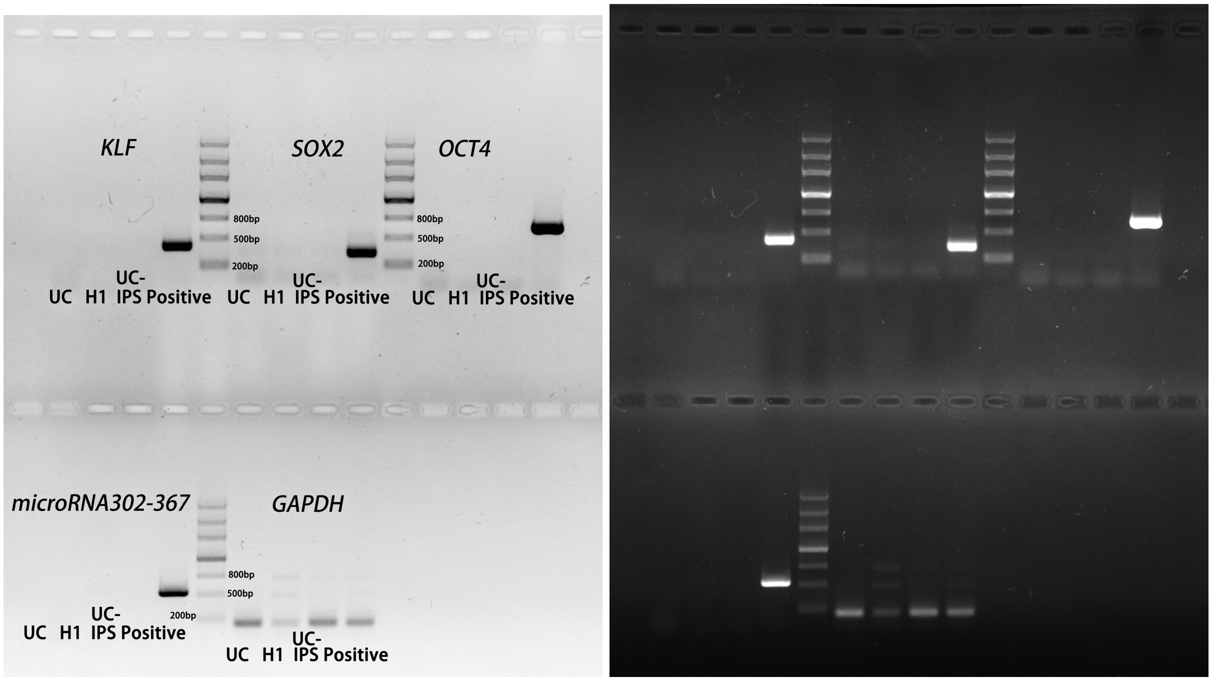

(a)

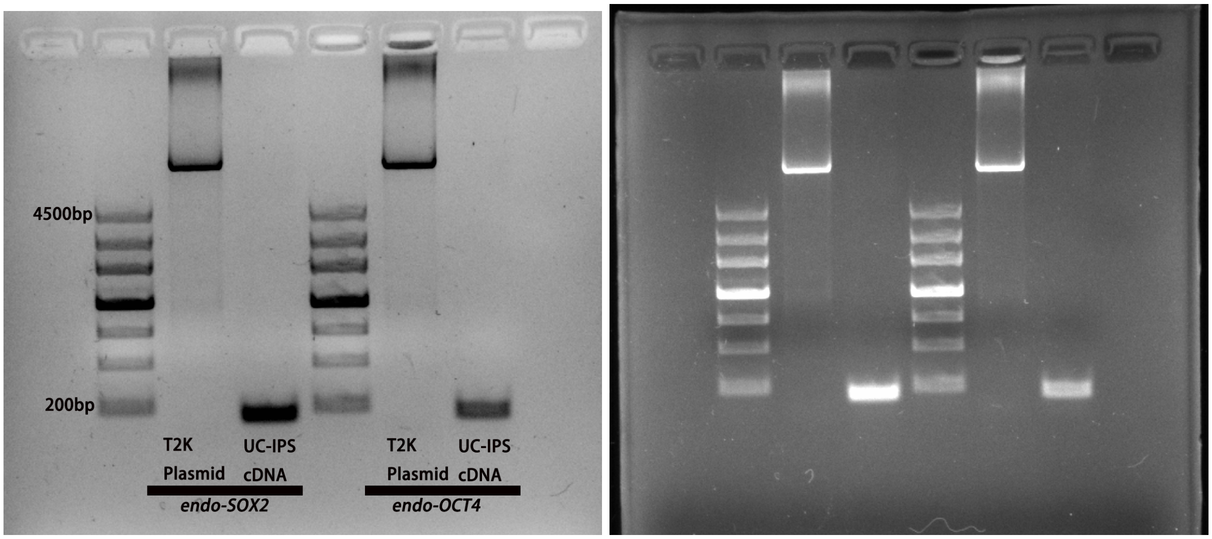

(b)

Figure S1. (a) PCR detection of exogenous eipsomal DNA in UC-iPSC. Uncropped full-length gels is shown in right. (b)PCR identify endogenous gene primer. pEP4EO2SET2K(T2K) plasmid was used as the negative control and the UCiPS cDNA used as the positive control. Uncropped full-length gels is shown in right.

Table S2. The primer used for genomic PCR and QPCR

	Gene name	Primer Name	Primer Sequence
For QPCR	endoOCT4	endoOCT4-F<br>endoOCT4-R	CCTCACTTCACTGCACTGTA<br>CAGGTTTTCTTTCCCTAGCT
	endoSOX2	endoSOX2-F<br>endoSOX2-R	CCCAGCAGACTTCACATGT<br>CCTCCCATTTCCCTCGTTTT
	NANOG	Nanog-q2-F<br>Nanog-q2-R	AAGGTCCCGGTCAAGAAACAG<br>CTTCTGCGTCACACCATTGC
	GAPDH	GAPDH-F<br>GAPDH-R	TGCACCACCAACTGCTTAGC<br>GGCATGGACTGTGGTCATGAG
For PCR	exo-OCT4	Oct4-SF1<br>IRES2-SR	AGTGAGAGGCAACCTGGAGA<br>AGGAACTGCTTCCTTCACGA
	exo-SOX2	Sox2-SF1<br>SV40pA-R	ACCAGCTCGCAGACCTACAT<br>CCCCCTGAACCTGAAACATA
	exo-KLF4	Klf4-SF1<br>SV40pA-R	CCCACACAGGTGAGAAACCT<br>CCCCCTGAACCTGAAACATA
	exo-miR-302/367	pCEP4-1627F2<br>pCEP4-C1306R	TTTCCAAAATGTCGTAATAACCCCG<br>CTCCCAAAGAGTCCTGTTCTGTCCT
	GAPDH	GAPDH-F<br>GAPDH-R	GTGGACCTGACCTGCCGTCT<br>GGAGGAGTGGGTGTCGCTGT

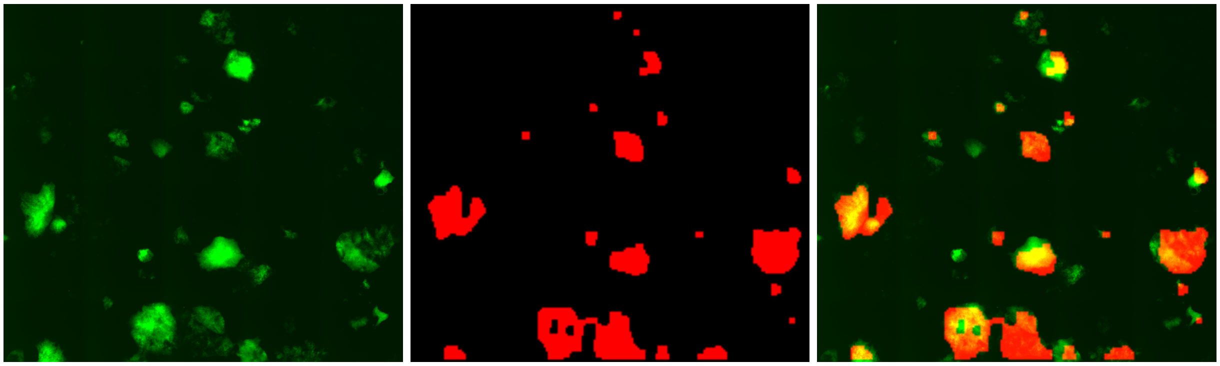

Figure S2. Test 2 and 3 of Mouse iPSC reprogramming detectin result.

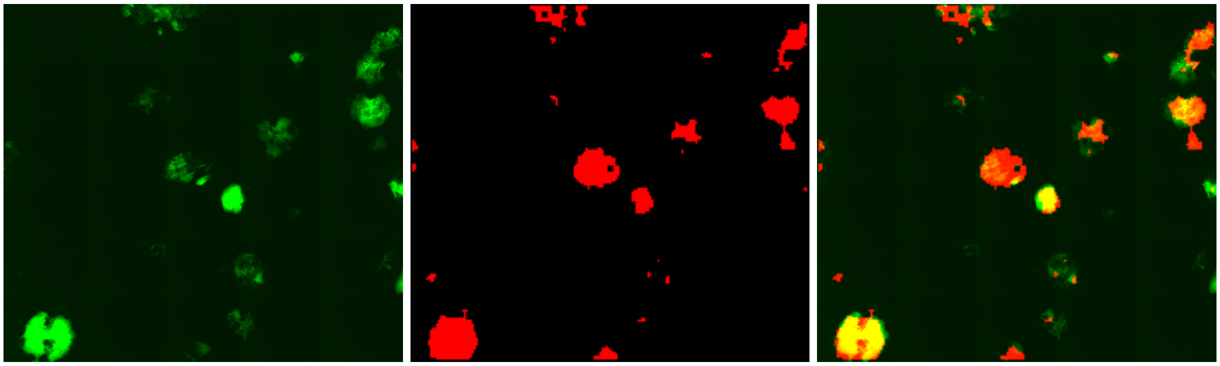

Figure S3. Test 2 and 3 of Mouse iPSC reprogramming detectin result.

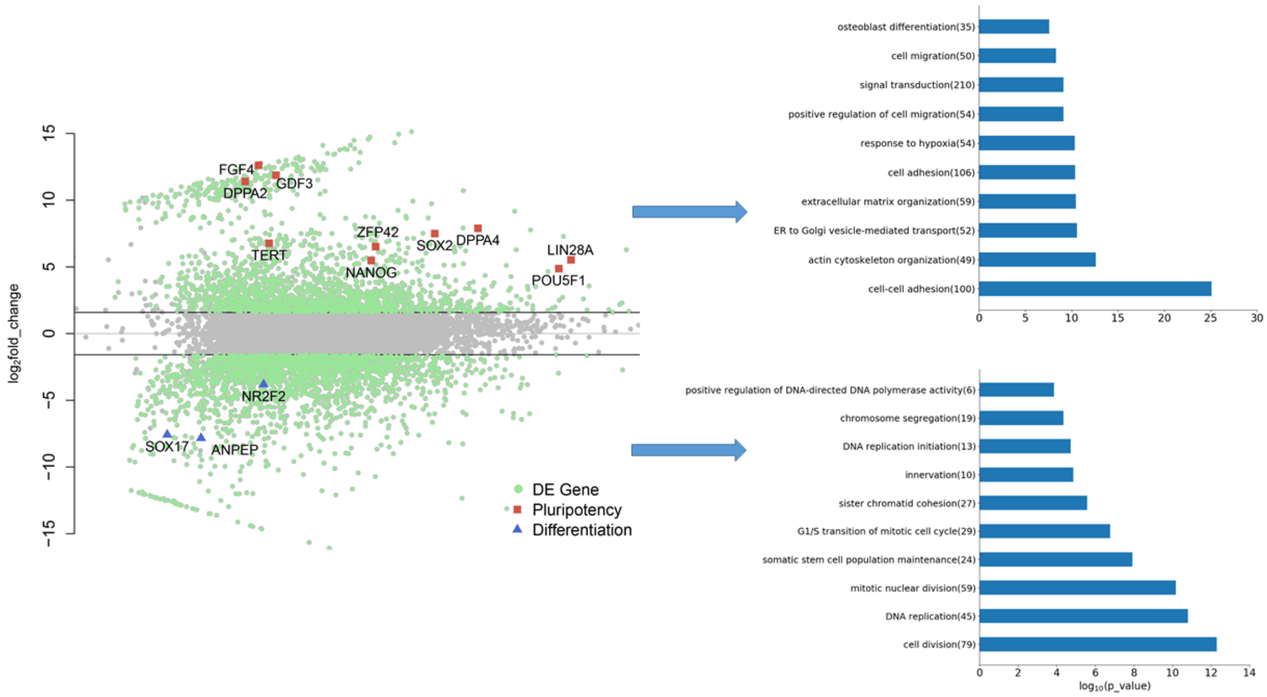

Figure S4. Differential gene expression profile between H1 (above) and UCs (below). The differentially expressed genes (red) are those with an adjusted P value 0.05 and fold change 3. Right figure shown functional annotations of genes differentially expressed between H1 (top) and UCs (bottom). Gene ontology (GO) was performed by DAVID, and enriched GO terms (biological processes) for each cell type are plotted with $-log_{10}$ of the adjusted P values.
